# A child and youth care approach to meeting the needs of children with visual impairments: A case study exploration

**DOI:** 10.4102/ajod.v15i0.1851

**Published:** 2026-03-20

**Authors:** Lydia Kankya, Rika Swanzen

**Affiliations:** 1 Faculty of Humanities, The Independent Institute of Education, EMERIS, Ruimsig, South Africa; 2 Ministry of Health, Office of Minister of State (Primary Health Care), Kampala, Uganda; 3 Faculty of Humanities, The Independent Institute of Education, EMERIS, Waterfall, South Africa

**Keywords:** child and youth care theories, needs of children with visual impairment, SDG targets 4.5 and 4.8, home and professional caregivers, use of daily life events

## Abstract

**Background:**

As indicated by the ‘interdisciplinary turn’, a multidisciplinary approach is critical to meet the needs of children with visual impairments (CWVIs). A less explored discipline, however, is that of the child and youth care (CYC) profession. The Circle of Courage Model and Being, Interpreting and Doing (BID) framework were used to design a semi-structured interview for caregivers of CWVIs.

**Objectives:**

A qualitative study was conducted to obtain an understanding of the CYC needs of CWVIs. The study aimed to obtain this understanding within the child’s school and home microsystems in order to identify challenges, gaps and contributions in meeting the CYC needs of CWVIs.

**Method:**

A qualitative case study design was followed with the semi-structured interview schedules derived from the mentioned CYC models. Fifteen caregivers were interviewed telephonically, comprising seven home caregivers and eight professional staff members. Interviews were transcribed and subjected to thematic analysis.

**Results:**

Six themes and nine sub-themes emerged from the analysis. A key finding is that the caregiver’s intentional approach to strength-focused development allows for holistic growth and self-confidence in CWVIs. The role of the caregiver is perceived to play an important role in creating ‘integral environments’ that cater for the CYC needs of the CWVIs.

**Conclusion:**

Findings from the case study confirmed the relevance of the CYC approaches to the meeting of needs and a context specific approach to activity programming. Recommendations are provided for the caregiver, the school, the CYC profession and society. A contribution of the findings is the unpacking of the needs of CWVIs within an ecological and activity context.

**Contribution:**

The results of the study provides insight into the understudied role of CYC in working with children with disabilities in an African context. It accentuates the importance of inclusive modes of communication on societal level, and resources required to ensure equal access to educational services. The article further contributes to the CYC knowledge base and meeting the United Nations Sustainable Development Goal (SDG) 2030 Agenda, targets 4.5 and 4.8, to ensure inclusive and equitable quality education and promote lifelong learning opportunities for all.

## Introduction

Sajan (n.d.:13) indicates that children with non-correctable vision problems who meet criteria such as low visual activity, visual field limitation and progressive eye disease of cortical visual impairment would require special education, because of the need for alternative models of instruction in reading and writing as well as affective education. ‘Visual impairment is estimated to affect 1.3 billion people worldwide, 19 million of whom are children younger than 15 years of age’ (Kuld, Kef & Sterkenburg 2021:131). In the Global South, the population of persons with disabilities (PWDs) are known to encounter challenges in terms of physical, cognitive, psychological and socio-emotional development, hindering them from reaching their full potential because of, among others, lack of care, poor nutrition, poverty and stimulation for growth, nurturing and development dynamics (Singal, Lynch & Johansson 2019:n.p.). The parent–child relationship is an important factor in the development of children with visual impairments (CWVIs), with a causal link to or comorbidity with cerebral visual impairment (VI), cerebral palsy, intellectual disability and autism spectrum disorder (Kuld et al. 2021:138).

Pederson (2016 in Lewis 2025) termed the ‘interdisciplinary turn’ to depict that no single discipline can have all the answers and that specialists need to move out of disciplinary silos to face complex and multidimensional global challenges. In South Africa (SA), Child and Youth Care (CYC) is a profession recognised by the SA Council of Social Service Professions. As such, CYC practitioners:

[*W*]ork with children, youth and families with complex needs … in a variety of settings such as group homes and residential treatment centres, hospitals and community mental health clinics, community-based outreach and schools-based programs … [*CYC*] workers specialize in the development and implementation of therapeutic programs and planned environments and the utilization of daily life events to facilitate change. (Stuart 2013:6)

Approaching the exploration from a CYC perspective implies that the meeting of the needs of CWVIs is studied within their main *lifespaces*, as elaborated upon below. The research question is: What are the needs of CWVIs from a CYC perspective? The sub-question explored the ways in which caregivers meet the CYC needs of CWVIs. This article offers insight from a case study conducted at a government school for the partially sighted and blind children in Gauteng, South Africa.

### Theoretical framework

The profession of CYC:

[*C*]onsiders development not from a chronological perspective but rather from a capacity perspective … the CYC developmental perspective is focused on confidence building around the demonstrated capacities of the young person or a family or even a community in order to aspire to further accomplishments. (Garfat et al. 2018:30)

A key concept in CYC, which is derived from an ecological-development approach, is ‘Lifespace work’. VanderVen (1991:n.p) refers to the fundamental nature of the lifespace concept as, ‘[*t*]he way we arrange the space of these interactions that connotes how well we care, and inexorably influences the significance of the caring message being given’. Child and youth care consistently aims to enhance the quality of life in holistic ways, creating spaces that meet the developmental needs of children, youth and families. This ‘Lifespace work’ concept also demonstrates the importance of the provision of day-to-day life experiences or daily living skills as the responsibility of caregivers or significant adults in a child’s life (Tapp, Wilhelm & Loveless 1991:118). This is relevant for the importance of CWVIs being provided with both living and working opportunities that foster independence – particularly in school, at home and within the community (LaVenture 2007:139). For CYC practitioners, emphasis is placed on the co-created relationship and meaning-making with the child, and this is organised through the set of universal development needs as identified from indigenous cultures such as the ‘Circle of Courage’ (Swanzen 2024). Another practice framework that represents the focus of the CYC profession is ‘Being, Interpreting and Doing’ (BID), as discussed hereunder.

### Circle of Courage Model

Firstly, the Circle of Courage Model (CCM) is embodied with four universal needs which enforce the positive development and encouragement of young people (Gharabaghi & Charles 2019:219). These comprise generosity, belonging, independence and mastery (Starr Commonwealth n.d). Mortimer Adler, a philosopher, labels these as ‘absolute needs’ that cannot be compromised, otherwise resulting in a ‘toxic environment’ (Brokenleg & Van Bockern 2003:23). This form of ‘toxicity’ is associated with the four seeds of discouragement specifically, which include destructive relationships, climates of futility, learned irresponsibility and lack of purpose (Stepney 2017:129). The CCM offers a depiction of universal needs, and if these are frustrated or not met, problems are likely to occur (Brendtro, Brokenleg & Van Bockern 2005:131), as opposed to the balanced circle that will enable the development of strengths and positive life outcomes. The CCM is elucidated through the specific inclusion of outcomes as per Table 1.

**TABLE 1:** The Circle of Courage Model: A paradigm for inclusive schooling.

The four dimensions of the CCM	Outcome
1. Belonging	Experiencing personal development, achieving social competence, having friends, forming and maintaining relationships, getting along with others and being part of a community.
2. Mastery	Being able to communicate, becoming competent in something and reaching one’s potential.
3. Independence	Engaging in problem-solving, assuming personal responsibility and accountability for decisions, having confidence to take risks and being a lifelong learner.
4. Generosity	Exercising social responsibility, being a contributing member of society, valuing diversity, being empathetic and being a global steward.

*Source*: Thousand, J. & Rosenberg, R.L., 2013, ‘The evolution of secondary inclusion’, in M. Nind, J. Rix, K. Sheehy & K. Simmons (eds.), *Curriculum and pedagogy in inclusive education: Values into practice*, pp. 164–176, Routledge, Oxon

CCM, Circle of Courage Model.

### The being, interpreting and doing framework

**FIGURE 1:**
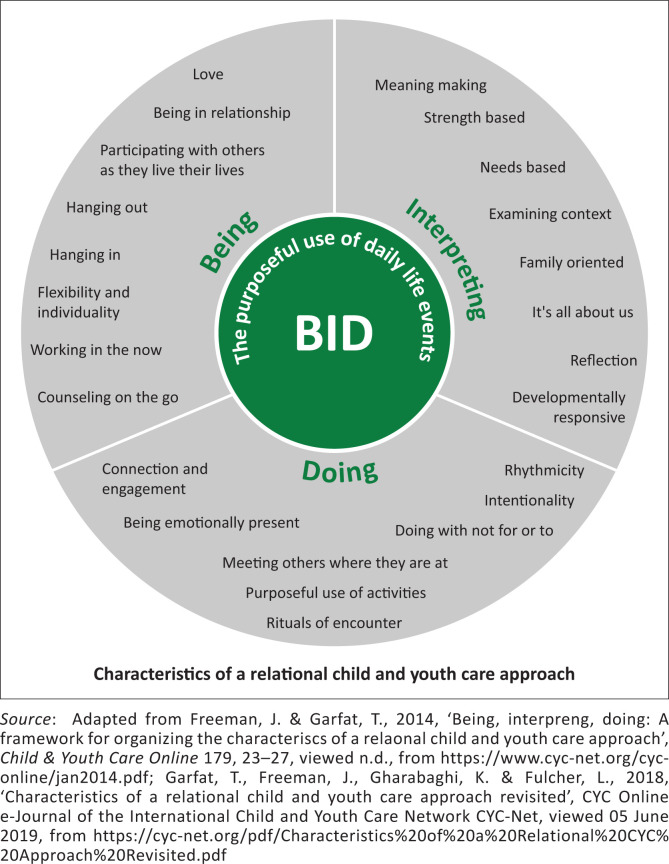
The being, interpreting and doing framework.

Secondly, the BID framework consists of 25 characteristics that are representative of the profession of CYC (Garfat et al. 2018:7). These characteristics demonstrate the relational dynamics within an environmental setting and are all-encompassing of cultural spaces, geographical locations and experiences (Garfat et al. 2018:7–9). The framework comprises three interconnected anchors: (1) love – classified under the category of *Being*; (2) meaning-making – classified under the category of *Interpreting*; and (3) connection and engagement – classified under the category of *Doing*. This framework aligned well with the research study, as it recognises that all humans possess individual strengths and that development or related challenges do not occur consistently in all areas of potential (Garfat et al. 2018:30). Figure 1 depicts the BID framework containing the 25 characteristics embedded within CYC practice.

From the 25 characteristics, the following were found to be relevant to this study, namely, love, developmentally responsive, needs-based, hanging out, purposeful use of activities and rituals of encounter. Including all characteristics was not feasible for this study, and the researchers therefore selected those most likely to consider developmental needs. Shaped through the concepts from the CCM and BID framework and considering the importance of caregivers in the well-being of CWVIs, the study focused on understanding and interpreting their needs within a CYC-informed lens. In the conceptualising of the study, the ecological theory by Bronfenbrenner and the hierarchy of needs by Maslow were consulted as related concepts. These theories were found to be complementary to the previously discussed frameworks, but were not specific enough to the relational approach CYC has to practice and are therefore not included in this article. The following section highlights the research methodology used in this study.

## Research methods and design

The focus of the study was delineated to the needs of CWVIs attending the school selected as the case study, regardless of the origin of the VI. Participation of the home care givers and professional staff was voluntary. The choice of this school allowed for the selection of various VIs; for example, blindness and partial sightedness – inclusive of oculomotor nerve palsy, nystagmus and all types of partial vision and colour blindness. Farquhar (2012:10) best describes case studies as ‘a rich multidimensional holistic picture of the situation’. The key strength of this approach is related to the fact that the researcher has minimal authority over events that are real-life-based (Yin 2003:1). One can also note the following key factors of such a research design (Simmons 2009:69):

- The need to understand programmes and policies through the perspectives of those who interact with them.
- An orientation to be educative is an interactive social process with the aim to comprehend the lived experience of the programme.
- Acknowledging that the interpretation of the experiences of the programme or aspects of the individuals’ lives occurs in specific socio-political contexts.

This exploratory research design approach permitted the researcher to deeply investigate the nature of a topic at hand (McNabb 2010:96). The case study method is context-specific and considers situational knowledge; the data cannot be transferred to an alternative setting nor generalised or replicated (Heinzl, Buxmann & Wendt 2011:121), and this is an acknowledged limitation of the study. However, in similar contexts to the case study, the findings may have some transferability, and it served its purpose to generate recommendations for CYC practice relating to a needs-based intervention for CWVIs. The aim of the study was not to obtain the perspective of children on their needs being met, and this will be important for further studies.

### Data collection and analysis

The CCM and BIDS frameworks discussed earlier formed the bases for the development of the semi-structured interview guide for both purposively sampled respondent groups. In 2020, social interaction was prohibited because of the global coronavirus disease 2019 (COVID-19) pandemic. The planned data collection method had to be adapted from in-person semi-structured interviews to telephonic semi-structured interviews. Audio-recorded telephonic interviews were conducted with 15 caregivers, all affiliated with the school. A purposive sampling technique was used to ensure those best informed were interviewed. The caregivers comprised two groups, namely, the professional staff of the school and parents or HCs of the children from the school. The inclusion criteria of the two sampled groups are provided in Table 2.

**TABLE 2:** Sampling criteria.

Criteria for professional staff (PS)	Criteria for home caregivers (HC)
Must be employed at the case-studied school	Have a child attending the case-studied school
Be within the age range of 20 years and older	Be within the age range from 20 years and older
Represent different ethnic origins	Represent a different ethnic origin
Provide expertise and nurturing for CWVIs as per the selection of categories	Nurture a CWVI as per the selection of categories
Represent one of the different areas of expertise in relation to the work of VI. Examples include principal, social worker, head of department, occupational therapist, psychologist, orientation and mobility specialist	Either be the biological parent, adoptive parent, foster parent, paternal uncle and/or aunt, maternal uncle and/or aunt, cousin (or of any family relation), in-law
Work with children of different age groups within Grades R to 12	Care for children of different age groups and from Grade R to Grade 12

CWVI, children with visual impairments; VI, visual impairment.

The sampled groups represented the two ecological microsystems of CWVIs, namely, their home and school environments. A thematic analysis was adopted as it enabled a thorough review of data (Nowell, Norris & Moules 2017:2). The six steps are captured in Table 3 (Maguire & Delahunt 2017:n.p).

**TABLE 3:** Thematic analysis steps.

Thematic analysis step	Description of task
Familiarising yourself with data	Thorough examination of the transcripts and making notes on first impressions and finding missing data.
Generating initial codes	Organise data in a meaningful and systematic manner, assigning codes to the data. Codes are generated on the basis of what is being said with consideration of the purpose of the study.
Searching for themes	Examines and categorises the codes under descriptive themes, taking into account similarities and differences.
Reviewing themes	Reviews the themes and ensures the data carry the weight to support the theme and fit within the context.
Defining and naming themes	Identifies pattern relationships, the significance of themes, sub-themes, their links and relation to the main themes.
Producing the report	The researcher begins to draft the report after completion of analysis.

*Source*: Adapted from Maguire, M. & Delahunt, B., 2017, ‘Doing a thematic analysis: A practical, step-by-step guide for learning and teaching scholars’, *All Ireland Journal of Higher Education* 9(3), 3351–33514

To verify the findings, field notes made during the telephonic interviews and reflexive journaling during the analysis of the code descriptions were created, as well as frequent consultations with the supervisor, in order to limit potential biases and ensure all views were considered. Both authors hold the relevant training to interpret the sharing of experiences that may be emotionally laden. Using Excel sheets as an audit trail and through repeated reviewing of keywords and considering the nature and repetition of each emerging theme, main and sub-themes were labelled and are described in the next section. The reviewing of the themes considered whether they spoke to needs within the CYC-informed concepts.

### Ethical considerations

Ethical clearance to conduct this study was obtained from the Independent Institute of Education IIEMSA Ethics Committee (Project no.: IIE MSA-EC-0089-2019) and the Gauteng Department of Education (No.:8/4/4/1/2). Approval for the study was also obtained from the relevant government department responsible for the school, including authorities from the school governing body. Participants signed the required consent forms, and their names were replaced with pseudonyms in the interview transcripts. Data were stored securely in line with the institution’s policy, and a summary of results was provided to the Gauteng department as part of the condition for their approval of the study.

## Results and discussion

Fifteen semi-structured interviews were conducted, comprising seven HCs and eight professional staff members (PSs) of CWVIs. The eight PSs consisted of one head of department, one occupational therapist, one orientation and mobility practitioner, one principal and four educators. Their ages ranged from 30 years old to 60 years old. The HC sample consisted of three fathers and four mothers, also ranging between the ages of 30 years old and 60 years old.

The first five themes reflect the CYC model and framework used to unpack distinct perspectives on the needs of CWVIs. Where a clear alignment was found between the theoretical concepts used and the themes or sub-themes, the term was added in italics within the heading or sub-heading. Supportive quotes from the caregivers are also added in italics within the sections. The themes identified from the findings of the research study involve the following:

### Family and community membership (*Belonging*)

Belonging enables an individual to experience a sense of purpose, inclusion, equality, acceptance and, most importantly, love. Findings showed that belonging has four layers, namely, equality of human rights, accessibility and provision in public spaces, exposure and integration, and trust, laying a strong foundation for meaningful relations with people.

#### Emotional care (*Love*)

‘Emotions are an integral part of what it means to be human and to live a human life’ (Jacobsen 2022:n.p.). Every human being is prone to experience emotions on a daily basis in their varying lifespaces, for example, in friendships and relationships, workplaces and at home. They define how we conduct ourselves as individuals, define ourselves, experience life, form relations with others and organise society (Jacobsen 2022:n.p.). In relation to CWVIs, emotions are expressed through physical touch and conversational means. It is noticeable that love is linked to integration for the CWVIs:

‘So I think for me the biggest that children need from their close ones is that kind of emotional and mental support to you know … For example, we visit out friends and they see the kids not playing with my son and they will actually go tell the kids “Go and play with him.” … that’s the kind of support I think they need.’ (HC2, 49 years old, male, self-employed)

‘Okay, we promote that sense of belonging by showing the world that these people can make it. We have a programme whereby we also take them to the streets, we train them openly. So, it’s part of making the world know that they exist, and they have the same right of walking as everyone. So, by taking them to the outdoor trainings – like in your malls – you train them in the malls how to use your escalators, how to go shopping, how to use the restaurants that type. So that also incorporates and shows the world at large that these people belong here with us.’ (PS7, 35–40 years old, female orientation and mobility specialist)

Caregivers revealed CWVIs consider the school as the ‘safe haven’ to either seek love they lack at home or to reconnect with those who are ‘similar’ to them. In the absence of this need, caregivers attribute this to difficult circumstances and socio-economic and family household dynamics, such as lack of father figures, child-headed households or single-parent households, which negatively impact the child’s behaviours and choices. Koelewijn et al. (2021:492) confirm that daily care from staff and managing tension among residents of a centre form part of a safe environment for the CWVIs. A concerning finding was that the majority of CWVIs lack father figures in the home, often resulting in feelings of abandonment. Respondents also raised issues relating to minimal love resulting from socio-economic circumstances and family household dynamics such as single-headed households and child-headed households. Caregivers also associated a lack of love with negative behaviours such as bullying, seeking love in the wrong places, craving attention and misconduct.

#### Membership, family practices and societal perceptions (*Rituals of encounter*)

It emerged that love is expressed through inclusion, as this results in a sense of belonging and eliminates feelings of discrimination:

‘So, you try and give them experiences-not only academic but on the sports field and then on obviously social gatherings – a simple thing like a school dance, where they will have music and they will dance and they will learn to socialise.’(PS4, 50 years old, female, head of department)

‘So, we need to come up with a way that we involve them in almost everything that we do.’ (PS5, 40 years old, male post-level educator)

‘… these kids need to be active in the social living. They need to get out and they need to socialise with specific people … they don’t want to be treated like they are visually impaired … their needs would be … to be handled as if they are normal.’ (HC4, 39 years old, female, non-employed)

Inclusion implied a desire for the children to be regarded in the same light as other children by the legislative sector, provision in public services (voting, grocery stores and bank ATMs), and removal from spaces of confinement (especially the home environment):

‘[*T*]hey need to know that those people will be able to help them in any way so they going to need to have a trust in the people … to actually help them, you know mobility wise.’ (HC4, 39 years old, female, non-employed)

Such inclusion facilitates a child’s overall well-being, confidence and integration in society, and meets their need to belong. As expressed by the respondents, the ability to relate and connect is a satisfying and reassuring feeling for CWVIs that he or she is normal:

‘You have to be sensitive for cultural differences … you do have a diverse group of learners and as teachers, we have to encourage each learner to accept his diversity and where he is-be proud of where he is or what his or her culture is … we will have a Cultural day where learners are encouraged to dress according to their culture-they love it! And they would then also talk … In my culture, this is what we do, these are my traditions, these are our beliefs et cetera.’ (PS6, 55 years old, female, principal)

‘[*I*]t’s that reciprocal respect thing … I see you and I see your mannerisms and your culture, and you see me and mine and we learn to respect one another – I think that’s acceptance … and self-realisation.’ (PS4, 50 years old, female, head of department)

Individuals are presented daily with challenges to their personal and cultural safety, simply by living in a community (Glisson, Dulmus & Sowers 2012:245). Gharabaghi and Charles (2019:150) discuss this with reference to the term ‘rituals of encounter’, which is interpreted as the practitioner’s role to engage with the client in meaningful ways that observe cultural differences. These cultural considerations can also be seen in the example of the Dialogue in the Dark exhibition (a physical and intellectual tour), in which visitors were presented the opportunity to gain insight into the world of the blind in order to encourage increased understanding (Gokcigdem 2016:68).

With regard to child-rearing, it was learnt that CWVIs are naturally accepting of different cultures, languages and beliefs (referred to as religion or background by caregivers) beyond their home norms and are inquisitive to learn more about them. This interest is a significant contributor towards embracing diversity, cultural recognition, acceptance, belonging and integration into society at large and with fellow peers in the school setting:

‘[*W*]e need to understand our kids … how our kids are learning and what they are learning and how to support them … I think we need to first be interested in our children and just know they … are normal kids and they can achieve whatever they want.’ (HC3, 38 year old, female, employed)

Common practices identified within family households included respect, togetherness, compromising, love, faith and openness towards foreign cultures and beliefs. With reference to the above list, several HCs agreed on the importance of engraving faith-based practices, for example, prayer, as a means of uplifting the child’s spirits, reassuring the child of his or her identity (avoiding developing a victim mentality) and knowing that there is hope in life. This is in line with the CYC developmental domain of practice milieus that considers the family as a microsystem, specifically within the cultural aspect that guides moral and religious identity (Stuart 2013:88–89).

A key finding in societal perceptions of normality was the fact that CWVIs desire to be seen as able and included:

‘You would find that with the partially sighted specifically … children generally reach their developmental milestones as normal. Unless there’s an added diagnosis (comorbidity).’ (PS8, 40 years old, female, occupational therapist)

‘They don’t understand why there are specific rights for children with VIs, or you know children with physical disability or anything like that … they know their rights but they don’t understand why it has to be different and why they have to be you know like sectioned off to the normal child who is the same age.’ (PS3, 30 years old, female, teacher)

From the opinions shared by the two groups of caregivers, it is evident that societal suspicions and negative connotations are an undeniable reality; the majority of the caregivers share the questions asked when in public and the uncomfortable interactions that children encounter in social settings, which can hinder social integration.

### Empowerment to achieve in all aspects of life (*Mastery*)

Echoed by a caregiver:

‘These children have got a hunger inside of them to achieve goals … they want to go out, they want to show you that they can accomplish. They have got a big hunger to … get rewarded for something. It’s like they have got this extra thing inside them to show the world, “this is what I can do.”’ (HC4, 39 years old, female, non-employed)

Therefore, in order to supplement this need, the HCs indicate how they introduce and inform the children of positive role models who have reached greater heights. The school also enforces this through their assembly sessions, in which former students address the learners.

The majority of caregivers note that this need, as not just a one-time event, enables children to discover their ‘inner talents’ to thrive on what they do best and gives them a place and identity to pursue something they are passionate about. This aspect is directly linked to character development of CWVIs, fostering participation in activities that align with interests, risk-taking, self-discovery, experiential learning and developing passion and confidence. Life principles are at the core of caregivers’ hearts to pass onto the child. This is in the form of daily *conversational teachings* on life responsibilities and personal protection. Interestingly, the respondents argued that CWVIs are more self-driven in nature and competitive, particularly because they know they require this more than the sighted. Caregivers note this need is greatly impacted by child-rearing practices that determine the child’s outlook and attitude towards life. They also mentioned the influence of the child’s personality, supported by Messer (1993:171), who relates temperament to young people’s inspirations regarding their activities, life goals and social aspects. Koelewijn et al. (2021:490) confirm the need expressed by caregivers to know the person their family member is, in order to focus on what is important to them.

### Orientation and mobility training towards enabling environments (*Independence*)

Independence provides a platform for an individual to be self-sustainable, and literature reveals that some children may be denied this opportunity or even overly protected by his or her parents as a result of high anxiety levels (Fielder, Best & Bax 1993:126). Catering to the need for mobility permits a child to imagine and experience the world through freedom of movement. It allows a child to exercise his or her independence and also grants privacy:

‘Oh they are wonderful! they move … I think quicker than seeing learners … if you look at the foundation phase, they are running the stairs …! They are playing on the playground, if we have visitors or we train a lot of institutions, and when they come and visit us they say, “but they can’t believe it’s blind learners” and we say that they are normal learners, they play, they go to their areas etc, but then again orientation and mobility-that is the key factor-because that will make the child not afraid to be able to move around and to play et cetera.’ (PS6, 55 years old, female, principal)

‘We look especially at orientation and mobility – that must start obviously – it’s an essential part of VI-their orientation and mobility because these learners will need to identify or “see the world” whilst they are moving.’ (PS2, 54 years old, female, teacher)

This skill is taught in gradual steps according to age appropriateness, capabilities and developmental stages, which are directly impacted by socio-economic circumstances:

‘I guess just having the necessary equipment and resources … to be able to be mobile and independent … As much as they need a buddy … a buddy that holds and guides, it’s important-one of the key things is the ability to have their own space and privacy because you don’t want to go to the toilet with a buddy for instance. So again, having the right resources to be able to move around on your own is very important.’ (HC1, 40 years old, female, self-employed)

According to caregivers, the sense of direction and mobility is developed gradually through extensive training both at home and in school, providing orientation through assistive devices such as a cane.

Caregivers state that joint collaboration between the child and caregiver is quintessential for the child to grasp the necessary skills that eventually lead to independence. According to them, the accommodation of this need is realised through descriptive verbal cues and physical demonstration. The intentional encouragement of mastery and independence is supported by recent literature on *habilitation*, in which services are aimed at providing support with challenges affecting well-being and mental health, such as ‘enabling independence in daily tasks, participation in social activities, and developing self-confidence’ (Manitsa & Barlow-Brown 2022:1). Safety precautions are a relevant part of skills development, such as food preparation, because of the complexities involved in kitchen hazards. Despite these challenges, accounts from PSs show that in the hostels, children partake in chores such as dishwashing. In both settings, caregivers share the daily routines of self-care, such as bathing, making the bed, brushing teeth and grooming practices, which proves children can do things individually and have the capability to take care of themselves.

### Give-and-take principle (*Generosity*)

Generosity is a key universal need which adheres to the rule of reciprocity, ‘give and receive or take’, as revealed in the feedback from one caregiver:

‘First of all, you know we try to teach our learners that generosity is not only something that can be extended to them. That they can also contribute to society and community … We will for example, look at Cancer Day and we would ask them through the Life skills period also to participate on occasions like that, we also have for example, where our learners will visit an old age home and so we try to teach them. It’s not a question of holding your hand out and just accepting, you must also have the responsibility of giving back or also of being part of community – of being generous yourself.’ (PS6, 55 years old, female, principal)

Other caregivers described the pattern of this need, noting that the more a child may be in need of generosity, having seldom experienced it, the harder it is for the child to extend this act towards others, whilst others attributed this need to home values and upbringing:

‘[*A*]nd I think this is something that we need to learn as a society but obviously once again, that’s a very personal thing, it depends on your upbringing, were you taught to be self-centred or you taught to look at the needs of others?’ (PS4, 50 years old, female, head of department)

The observations from PSs revealed that this trait is naturally displayed by their students without being told:

‘I found that my son is actually very helpful towards other people and I think it’s just the culture from the school.’ (HC1, 40 years old, female, self-employed)

Some examples of these acts include the more mobile students assisting the teacher or fellow students in the classroom and assisting newcomers who are unfamiliar with the school environment. Other examples that display the children’s sense of collective responsibility include assisting a fellow student to locate a Braille machine which had been misplaced within the classroom and assisting fellow students by carrying their bags. Home caregivers expressed that generosity boosts a child’s self-esteem and provides them with a sense of pride and achievement for their positive contribution.

### Broad alternative development needs

Child development involves the gaining of skills within physical, cognitive, socio-emotional, language communication and sensory developmental areas, and these are influenced by familial and environmental factors (Heyns & Roestenburg 2021:2). It was highlighted that the developmental needs of CWVIs are similar to those of sighted children, although there are a few broad alternative developmental needs that were singled out as alternative needs of the CWVIs. These are identified under the following sub-themes:

#### Age differentiation in socialising (*Hanging out*)

Findings show that when it comes to socialising, CWVIs have the need to be understood, to be included, heard, accepted and noticed:

‘And it’s very important as well for them to hang out with their peers you see and enjoy whatever it is that kids enjoy these days.’ (HC7, 40–50 years old, female, employed)

The act of socialising is linked to the sense of belonging in which CWVIs establish their identity and membership in the space of hanging out. Caregivers note the need to socialise with others as highly beneficial to the child, as it meets their cultural needs; enables them to have a sense of belonging, love and acceptance; creates an atmosphere of comfort and authenticity in which one is able to express themselves; and equips the child with essential social skills for real-life situations. Another perspective raised by an HC in respect of hanging out sheds light on the fact that these children stop seeking validation at an older age as a result of a higher sense of self-value and ability in relating to others. However, an issue identified within this theme is the fact that some CWVIs tend to hang out with the elderly because of lack of integration, acceptance and uncomfortable questioning (or even bullying) from sighted peers in their age group.

#### Medical needs

In relation to the medical needs of a CWVI, HCs identify this need as one of their lifetime investments and responsibilities:

‘[*A*] they develop-one was already conditioned … in terms of medical attention, this is what you have to do for the rest of his life you know. Or eye check-ups or dermatologists, it’s second nature, it’s like me or you going for a flu or whatever you know.’ (HC7, 40–50 years old, female, employed)

Similarly, with a person who has a medical condition, the same applies to CWVIs. The medical needs of these children can be broad, as regular check-ups with relevant medical practitioners are required to maintain their overall well-being and address any sudden destabilisation in their health.

#### Assistive equipment

Children with visual impairments are dependent on assistive equipment, which enables their development and allows them to function on a daily basis with some level of mobility and receive a quality education similar to that of a sighted child:

‘[*T*]hey need to have a braille machine, you can’t teach them, or you can’t educate them actually without that.’ (HC4, 39 years old, female, non-employed)

‘The need is the right equipment to do it, and to be able to make the program available to teach them how to use that equipment. *Ah* like I told you, if a CWVI, doesn’t have a braille machine or a cane, how are they going to get through life?’ (HC5, 35 years old, male, telephone specialist)

Some of the most important tools caregivers identify are the cane, Braille machine and devices, such as a video magnifier for the classroom.

#### Sense of privacy (*Doing with and not for or to*)

The benefit of not doing for or to the CWVIs is that they can experience freedom of movement, the right to privacy, confidence and the ability to act autonomously, enabling transition into adulthood:

‘I suppose for me the one thing I’m taking easiest, the one thing I’m not really pushing so far is the ability to be independent to do a few things … for him to be totally independent living. I think for some assistance for him whether it’s technological or someone living with him.’ (HC2, 49 years old, male, self-employed)

Independence is acknowledged to be a gradual development process and only granted to the child with certain measures in place to ensure safety and avoid stretching a child beyond his or her capacity.

#### Participation in activities (*Purposeful use of activities*)

Caregivers contextualised this theme as a child partaking in ‘purposeful activities’, including those that may be ‘meaningless’ as purposeful to the child:

‘By participating in activities, it helps with their social development, it also helps with knowing “I can do this, I’m successful … it gives them the confidence for example, standing on a stage and singing. It gives them knowing, “I’m not different, I’m not disabled, I’m abled to do what any other child can do.” … [*O*]ur learners are very musical, they have wonderful talents and love music, they are always singing. So, if you foster and you cherish the activities that you can provide to them … you bring the outside world to the school.’ (PS6, 55 years old, female, principal)

“It should be something he can relate to. Okay … either he can hear, so it’s got to be something that some of his 5, 4 other senses can act … can read to, can hear it, can touch it can smell it or taste it. … [*I*]f we take him to go to a restaurant, and he can eat good food and he’s happy. Can he physically take part in it? Can he play? So, like when we went to that national park, he can’t … well animals don’t really make much noise they are a bit in a distance, he can’t hear them, he can’t smell them, he can’t see. So, all you’re doing is driving around in a car and other people are seeing things that he can’t see … so they got to be involved.’ (HC2, 49 years old, male, self-employed)

Caregivers contextualised this theme as a child partaking in ‘purposeful activities’, including those that may be ‘meaningless’ as purposeful to the child. The caregiver’s intentionality to ensure the child is proactive and provided with a wide range of opportunities enables experiential learning. For example, one caregiver shared how *Mecannos* (a toy model construction system) and outdoor ventures are means by which experiential learning opportunities are created. On the other hand, caregivers highlighted the lack of opportunities attributed to socio-economic circumstances.

A key requirement with activities is that they need to be age- and developmentally appropriate activities (*Developmentally responsive*) and should also be of interest to the child:

‘But the needs must first be identified, “What can the learner do? What can the learner not do?” considering the age, the grade and then you work from there-you work from there.’ (PS6, 55 years old, female, principal)

‘What is expected from a normal child to achieve at a certain age that’s the exact expectation that we should have for a CWVI. So, I think that CWVI should be able to just learn age-appropriate skills or … whatever chores or whatever that they need to do around the home or just for themselves and as a person you know.’ (HC3, 38 year old, female, employed)

‘The children have a right to education, a right to everything but if I take education as an example, if you take children whose socio-economic situation where they don’t have the right to education on their level, then it seems they are pushed into schools, they are not ready for them and then they fall behind in a way that sometimes they never catch up again. If they finally get to a school where they are prepared for CWVIs, they are so far behind and it takes them a much longer time to get there.’ (PS1, 42 years old, female, teacher)

This perspective is supported in the United Nations Educational, Scientific and Cultural Organisation (UNESCO)’s report (UNESCO 2020), which outlines the disproportionate education and discrimination disadvantage. The following point stated in the report aligns with the legal guidelines aspect identified in this study:

CWVIs (particularly those enrolled in schools) have little or no access to necessary assistive technology – for instance optical and non-optical low-vision aids for children with low vision and braille writing equipment and braille reading materials for children who are blind. (UNESCO 2020:13)

Participation in age-appropriate activities strengthens inclusion, which the CWVI has a right to, that permits the use of senses and manageability within their developmental capabilities. This is validated by one caregiver’s input, in which confidence-building and the abilities of the child are the two key factors to consider when planning the activities. It is evident from the findings that activities have numerous benefits when the child’s needs are catered for, such as a sense of community, achievement, pride, purpose, enhancement of social interactions, brain stimulation, consistency, physical health and strength, life skills, motivation and heightening of senses and confidence levels – active learning through doing:

‘[*B*]ut everything is not about enjoyment … I think an important thing here is relevance, do they find it relevant? Does it speak to them? Are they interested? … [*A*]cademic wise, I think sometimes they are forced to be interested even though they are not really - they realise they have to show interest because they have to pass. But very often as I said, these meaningless activities-what we would regard as meaningless, they would think those activities are wonderful.’ (PS4, 50 years old, female, head of department)

Another needs aspect laid out in the theme of participation addresses the importance of the interests of the child, which include specific outcomes and objectives set by caregivers in the planning of activities. Children with visual impairments desire to partake in activities that have the following characteristics: activities that fall within his or her interests, define and highlight personal strengths, are entertaining and, most importantly, have a tactile component or use other senses. It was found that a fair balance in the approach of activity planning is key and should aim to enhance the holistic development of the child to access the benefits from activities.

### Alternative tools for expression and learning

Child development involves the gaining of skills within physical, cognitive, socio-emotional, language communication and sensory developmental areas, and these are influenced by familial and environmental factors (Heyns & Roestenburg 2021:2). It was highlighted that the developmental needs of CWVIs are similar to those of sighted children, although there are a few broad alternative developmental needs that were singled out as alternative needs of the CWVIs. These are identified under the following sub-themes:

#### Boundaries and using the sense of touch to express emotions

Caregivers conceptualise boundaries in three ways, namely, generosity, implying the give-and-take principle, socialising and relationships:

‘If we talk about peer relationships, CWVIs will also need to know there are boundaries. They tend to be over enthusiastic sometimes when they are smaller. They also need to learn the Dos and Don’ts of socialising and being able to be in a relationship a peer relationship with the friends.’ (PS6, 55 years old, female, principal)

This is done through conversational teachings in which a set of safety guidelines are shared with CWVIs in regard to their relations with others in order to protect them from vulnerable situations. This initiative enables children to be aware that boundaries are mandatory and are required to be set in a way that they are able to assert themselves in the company of others. Caregivers use everyday experiences attached to the importance of humanity.

#### Echolocation as a learning tool

Caregivers distinguish between two age groups of CWVIs, in which the younger group prefers holding hands, pats on the shoulder, conversations or comforting, whereas the teen prefers conversations, showing the varying forms of demonstration of love:

‘They totally rely on touch and for you to teach them the things around them, they need to feel it … to touch it … to learn their environment.’ (PS1, 42 years old, female, teacher)

‘I think that the traits any CWVI … especially a younger age, is always touching. You know teaching at a visually impaired school-when we interview teachers, one of our vital questions is “How do you feel about your own physical space?” Because teaching at a school for CWVIs means learners are constantly touchy, but I mean that’s their way of experiencing, that’s their way of seeing.’ (PS6, 55 years old, female, principal)

Several caregivers expressed the importance of enhancing vision through tactile means. Home caregivers meet this need through clarifying questions for children after social interactions with people, introducing and equipping the child with knowledge through the introduction to concepts and environments for experiential learning purposes. In the context of the school setting, touch is used as tactile exploration to teach children about their environment and concepts, done through teaching the size of objects, learning about food, for instance, the size of a tomato or cucumber, using models for conceptualisation, such as escarpment, and equipping the child with knowledge of concepts such as the feel of coins and notes of money:

‘First of all, to find their own relationship into the space around them … as I said my child does echo location like a bat … and they must first find out what is the surroundings - everything around them - so that development need is definitely there … my child uses echo location and not a dog or a cane. But he uses echo location so he can sense when there is something in his way and he walks around but we also take care. We tell him if there is something that is in his way … I’m just looking-while he is feeling all over and even tend to understand … he is always touching everything and looking at everything, but they don’t understand that his eyes is now in his hands. So, the world needs to understand that.’ (HC6, 53 years old, male, factory CEO)

Caregivers identify ‘echolocation’ as a key need to get acquainted with their environment, leading to a sense of direction. This self-positioning comprises navigation and obstacle avoidance (Melcón & Moss 2014:10).

Harbin (2009), the World Health Organization (2024) and Disability Info South Africa (2025) express interchangeable views regarding the limitations encountered by people with VI in performing normal daily activities such as reading, driving, walking and socialising. These often lead to high dependence on third parties or the use of visual aids and other equipment (Harbin 2009:117).

Although most HCs noted that there is no difference between the expression of love towards the children, a PS did distinguish that a CWVI requires abundant love compared to sighted children. The caregiver related this to the non-visual aspect compared to the sighted child who is able to see a facial expression.

What the findings highlighted is that the caregiver’s intentional approach to strength-focused development allows for holistic growth, self-confidence and self-esteem. From the findings, it is evident that the school set-up has opened a new world of diversity to the learners. Home caregivers share how their children are now fluent in other languages and even native languages, which they could not speak prior to joining the school. The role of the caregiver is perceived to play an important role in creating ‘integral environments’ that cater to the various needs of the CWVIs. This *integral environment* comprises a wide range of interconnected segments (e.g. love, inclusion, assurance, personal space and education) that enhance the holistic development of the CWVIs. With regard to stimulating environments, findings show that these spaces should permit the following: individuality or identity, culture, supportive structures, equality, inclusion, personal interests, decision-making, and strengths and skills development. These findings also coincide with CYC principles of being in relationship and fostering the needs and capacities of children and youth (Paget 2016:8) and the creation of fulfilling life spaces to define the purposeful use of daily events (Garfat et al. 2018:12; Stuart 2013:295). A study that explored common terms from literature used with CWVIs found challenging and self-injurious behaviour to be a common subject term (Kuld et al. 2021:138). It is interesting that this study, which explored CWVIs from terms derived from CYC theory, did not result in significant concerns raised with negative behaviours (outside of it being caused by the lack of love), which confirms the strength-based approach embedded in their theory.

## Implications for practice

Judith Heumann, a disability rights activist, once said: ‘Disability only becomes a tragedy when society fails to provide the things needed to lead one’s daily life’ (Shapiro 2023:n.p.). Recommendations for the caregivers include the establishment of support groups and forums to allow for knowledge exchange and sharing of personal experiences and solutions. Deducing from the findings, a shared partnership between caregivers is required to facilitate the formulation and regular monitoring of an *individualised education programme* tailored to cater for each child’s needs. In meeting this need, the reorganisation of the placement of objects is a key initiative taken by caregivers. This is relevant in various settings, especially the classroom and home. For schools, the *adjustment in teaching pace* is also a key factor to enhance critical thinking, problem-solving and active, developmentally appropriate engagement. This would include the enlargement of computer screens, closed-circuit cameras, screens placed at the learners’ desks, enlarged print size, a decrease in group size and light sensitivity. Adaptations in the child’s environment play a crucial role in ensuring free and safe movement. Mainstream schools should be mandated to assimilate special needs within their operational means, that is, acquiring assistive equipment and ensuring access to multidisciplinary teams. For the profession of CYC, the understanding of the needs to be met also informs the *activity programming* roles of CYC workers, especially their use of daily life events for developmental and therapeutic aims. The synergy between the CYC concepts and the needs of CWVIs supports the contribution CYC can make to the multidisciplinary team.

Whilst this article broadens the understanding for CYC practitioners working with CWVIs, it also provides exposure to the profession in the disability sector. Further research on the role CYC plays in this sector is needed, especially around how they function as complementary to the medical and educational aspects involved in the care of children with disabilities provided by other disciplines.

On a societal level, increasing inclusion calls for the adjustment of the modes of communication used in public, such as having posters in Braille and auditory narration, especially on important public health matters. Updated statistics need to be used to plan for the building of schools needed for children with impairments or for inclusion programmes in mainstream schools. This strategy should include fundraising for the necessary equipment that will ensure CWVIs have equal opportunities. Such a commitment meets the CWVI’s educational need in terms of the U.N SDG4, in particular targets 4.5, which is to eliminate all discrimination in education, and 4.8, which states to build and upgrade inclusive and safe schools (United Nations Department of Economic and Social Affairs 2025). Being intentional about diverse integral environments, individualised learning plans and the adaptation of environments contributes to inclusive and safe schools, whilst developmentally responsive, assisted participation in activities, with a respect for boundaries and the encouragement of independence, will enable non-discriminatory, equitable and inclusive practices in the education of CWVIs.

## Conclusion

The contribution of this study lies in the exploration of the CWVI’s needs from the CYC perspective through the inclusion of caregivers from their school and home environments. The diversity among the population under study showcased varying situations, ranging from household structures, professions, cultures and the wide spectrum of VI represented, resulting in practical insights. This study aimed to explore the needs of CWVIs from a profession’s perspective that is not yet fully integrated into the multidisciplinary team. Including the CWVIs in a similar exploration will be a further worthwhile pursuit.

Findings confirmed the significance of care and belonging within the home and school environment systems in fulfilling the CWVI’s needs. The case that was studied showed progressiveness in terms of inclusive practices and in providing resources to enable the development of mastery, independence and generosity with their learners, whilst also responding to their need for belonging through integration and the demonstration of care. Caregivers expressed the desire for the CWVIs to be seen as ‘normal’ and not segregated, and that the needs and desires are similar to that of the sighted child.
